# Exosomal secretion of α-synuclein as protective mechanism after upstream blockage of macroautophagy

**DOI:** 10.1038/s41419-018-0816-2

**Published:** 2018-07-09

**Authors:** Natascha Fussi, Matthias Höllerhage, Tasnim Chakroun, Niko-Petteri Nykänen, Thomas W. Rösler, Thomas Koeglsperger, Wolfgang Wurst, Christian Behrends, Günter U. Höglinger

**Affiliations:** 1 Department of Translational Neurodegeneration, German Center for Neurodegenerative Diseases (DZNE), D-81377 Munich, Germany; 2 Department of Neurology, Technical University of Munich, D-81675 Munich, Germany; 3 Munich Cluster for Systems Neurology (SyNergy), Ludwig Maximilians University, D-81377 Munich, Germany; 4 Department of Neurology, Ludwig Maximilians University, Munich, Germany; 5 Institute of Developmental Genetics, Helmholtz Center Munich, German Research Center for Environmental Health, D-85764 Munich, Germany; 6 German Center for Neurodegenerative Diseases (DZNE) Site Munich, D-81377 Munich, Germany; 7 Chair of Developmental Genetics, Center of Life and Food Sciences Weihenstephan, Technical University of Munich, D-85764 Munich, Germany

## Abstract

Accumulation of pathological α-synuclein aggregates plays a major role in Parkinson’s disease. Macroautophagy is a mechanism to degrade intracellular protein aggregates by wrapping them into autophagosomes, followed by fusion with lysosomes. We had previously shown that pharmacological activation of macroautophagy protects against α-synuclein-induced toxicity in human neurons. Here, we hypothesized that inhibition of macroautophagy would aggravate α-synuclein-induced cell death.

Unexpectedly, inhibition of autophagosome formation by silencing of *ATG5* protected from α-synuclein-induced toxicity. Therefore, we studied alternative cellular mechanisms to compensate for the loss of macroautophagy. *ATG5* silencing did not affect the ubiquitin–proteasome system, chaperone systems, chaperone-mediated autophagy, or the unfolded protein response. However, *ATG5* silencing increased the secretion of α-synuclein via exosomes. Blocking exosomal secretion exacerbated α-synuclein-induced cell death.

We conclude that exosomal secretion of α-synuclein is increased after impaired formation of autophagosomes to reduce the intracellular α-synuclein burden. This compensatory mechanism prevents α-synuclein-induced neuronal cell death.

## Introduction

Parkinson’s disease (PD) is a neurodegenerative movement disorder characterized by the loss of dopaminergic neurons in the substantia nigra pars compacta (SNc) and the accumulation of the protein α-synuclein (α-Syn) in Lewy bodies or Lewy neurites in vulnerable neurons^1–3^. α-Syn is a small presynaptic protein which consists of three domains. Its physiological functions are still not fully understood^4,5^. Point mutations (e.g. A30P, G51D, A53E, A53T, E46K) of *SNCA*, the gene encoding α-Syn lead to autosomal-dominant PD^6^. Also, duplications and triplications of wildtype *SNCA* cause autosomal-dominant PD^7,8^. However, monogenic forms of PD are rare and most cases are sporadic^9,10^. Nevertheless, genome-wide association studies consistently found single nucleotide polymorphisms in *SNCA* as major risk factors for sporadic PD^11–13^. α-Syn can be degraded by various intracellular protein degradation mechanisms, including the ubiquitin–proteasome system (UPS)^14^, chaperone-mediated autophagy (CMA)^15^, or macroautophagy^14^.

Macroautophagy, from here on referred to as autophagy, is a highly complex process, which is initiated with the formation of a phagophore. Subsequently, the phagophore forms a double-membrane layer structure called the autophagosome, a structure responsible for engulfing unwanted proteins or cell organelles in the process of autophagy. Next, the autophagosome fuses with the lysosome to form the autophagolysosome, in which the actual degradation of imported cargo occurs^16,17^. Autophagy is perceived as a rather selective process^18,19^ with distinct subtypes of autophagy targeting cell organelles (e.g. mitophagy), or other intracellular structures (e.g. lipophagy, aggrephagy). Previously, we have shown that pharmacological stimulation of autophagy in Lund human mesencephalic (LUHMES) neurons protects from α-Syn-induced toxicity^20^. Here, we aimed to investigate the effects of autophagy inhibition on α-Syn-induced cell death. Surprisingly, we found that blocking of autophagy by silencing of autophagy-related gene 5 (*ATG5*), which is crucial for the formation of autophagosomes^21^, protected LUHMES neurons from α-Syn-induced cell death. We hypothesized that the cells are activating an alternative mechanism for α-Syn disposal upon impaired autophagosome formation. Therefore, we investigated the effects of *ATG5* silencing on other possible mechanisms to attenuate the accumulating cellular α-Syn burden, such as the UPS, chaperones, CMA, the unfolded protein response (UPR), and exosomal secretion.

## Results

### Silencing of *ATG5* rescued LUHMES cells from α-Syn-induced cell death

*ATG5* is a key player of autophagy and is essential for autophagosome formation. We investigated the role of *ATG5* in α-Syn overexpressing LUHMES neurons. Therefore, we overexpressed α-Syn by adenoviral transduction and silenced *ATG5* by siRNA transfection.

**Fig. 1:**
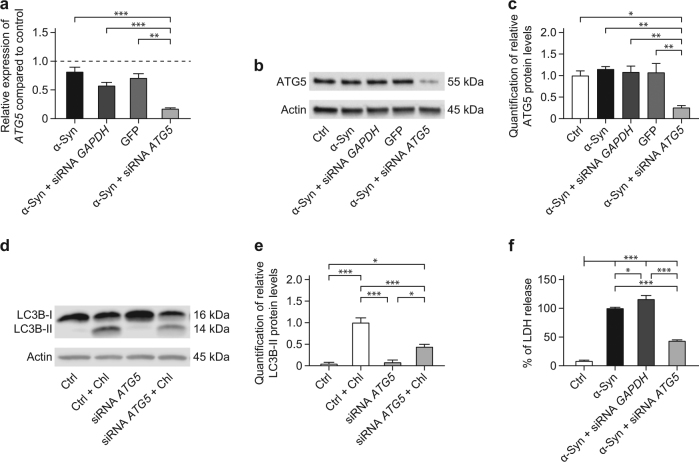
*ATG5* siRNA protects against α-Syn-induced toxicity. The effect of *ATG5* siRNA on *ATG5* expression, autophagosome formation, and α-Syn-induced toxicity was analyzed. **a** qRT-PCR for *ATG5* in cells overexpressing α-Syn, in cells overexpressing α-Syn and transfected with control siRNA against *GAPDH*, cells overexpressing the control protein GFP, and cells overexpressing α-Syn and transfected with *ATG5* siRNA, showing the silencing efficacy of *ATG5* siRNA on the mRNA level. The dashed line shows *ATG5* levels in naïve (untransduced, untransfected) cells as reference. **b** Representative Western blot for ATG5 protein in naïve control cells (Ctrl) and cells in the conditions reported in **a**. β-actin was used as loading control. **c** Quantification of ATG5 protein, normalized to β-actin, from Western blots as shown in **b**, showing the silencing efficacy of *ATG5* siRNA on the protein level. **d** Representative Western blot for the autophagosome marker LC3B in naïve cells (Ctrl), and cells transfected with *ATG5* siRNA, with or without chloroquine (Chl) treatment to block autophagosome–lysosome fusion. **e** Quantification of LC3B-II protein, normalized to β-actin, from individual Western blots as shown in **d**, showing that Chl increases LC3B-II in naïve cells more than in *ATG5* siRNA treated cells. **f** Quantification of lactate dehydrogenase (LDH) released into the culture medium as measure for toxicity. Data are expressed as percentage of α-Syn. *ATG5* silencing significantly reduced α-Syn-induced toxicity. Data in **a**, **c**, **e**, **f** are mean ± standard error from *n* ≥ 3 biological replicates. *p < 0.05, **p < 0.01, ***p < 0.001; one-way analysis of variance with Bonferroni’s post hoc test

α-Syn overexpression, a control siRNA against *GAPDH*, and overexpression of the control protein GFP did not change the levels of *ATG5* mRNA (Fig. 1a) and ATG5 protein (Fig. 1b, c) compared to naïve cells. However, *ATG5* siRNA in α-Syn overexpressing cells reduced the *ATG5* mRNA levels to 17.4 ± 0.02% (*p* < 0.001; Fig. 1a) and ATG5 protein levels to 25.8 ± 4.5% (*p* = 0.005; Fig. 1b, c) compared to naïve control cells.

We investigated whether *ATG5* knockdown changed the autophagic flux. As marker for autophagosomes, we used Western blotting for LC3B-II, which is generated from LC3B-I by phosphatidylethanolamine conjugation^22–24^. To differentiate between generation or degradation of autophagosomes, we added chloroquine, which inhibits the fusion of autophagosomes with lysosomes, resulting in an accumulation of autophagosomes, i.e. increased LC3B-II levels^23,24^. Expectedly, chloroquine increased LC3B-II levels in naïve control cells, showing that there was a regular autophagic flux (Fig. 1d, e). In cells treated with chloroquine, A*TG5* siRNA reduced LC3B-II levels (*p* < 0.001), demonstrating that *ATG5* silencing indeed reduced the generation of autophagosomes in LUHMES neurons.

The impact of *ATG5* silencing on intracellular α-Syn levels was monitored in the presence and absence of chloroquine, showing that *ATG5* silencing, chloroquine treatment, or a combination of both, had no significant impact on the intracellular α-Syn levels (Supplementary Figure S1).

Next, we investigated the effect of *ATG5* silencing on cell viability by quantification of LDH released into the cell culture medium (Fig. 1f). Transfection of naïve (untransduced, untransfected) cells with *ATG5* siRNA and of α-Syn overexpressing cells with control siRNA against *GAPDH* did not significantly affect cell viability. However, *ATG5* siRNA in α-Syn overexpressing cells reduced LDH levels to 43.32 ± 2.012% (*p* < 0.001), indicating that inhibition of autophagy by *ATG5* silencing protected the cells against α-Syn-induced toxicity.

**Fig. 2:**
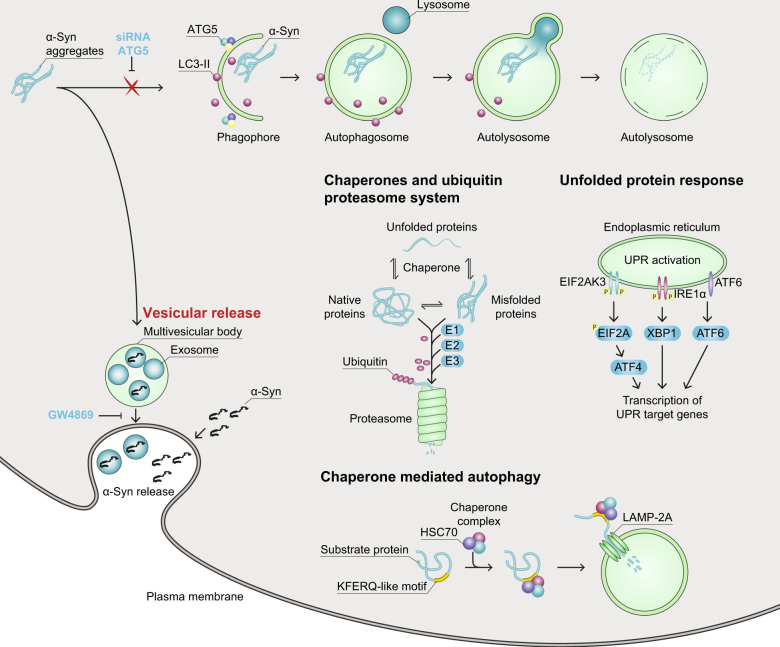
Potential cellular pathways to dispose of α-Syn aggregates. In macroautophagy, misfolded proteins and dysfunctional organelles are wrapped into a phagophore to form an autophagosome, which fuses with a lysosome to become an autolysosome for degradation of its content. *ATG5* knockdown inhibits the formation of the phagophore. Chaperones support the physiological folding process of newly synthesized or misfolded proteins and target irreparably misfolded protein for degradation. The ubiquitin proteasome system (UPS), degrades misfolded proteins for proteasomal proteolysis after labeling them with a poly-ubiquitin chain by successive action of ubiquitin activation enzymes (E1, blue), ubiquitin conjugation enzymes (E2, blue), and ubiquitin ligases (E3, blue). For chaperone-mediated autophagy (CMA), HSC70 and a chaperone complex recognize a KFERQ-pentapeptide motif of a protein and bind LAMP-2A in the lysosomal membrane to target the protein for degradation by lysosomal proteases. The unfolded protein response (UPR) is activated by accumulation of unfolded or misfolded proteins in the endoplasmic reticulum via three stress sensors (EIF2AK3, IRE1α, ATF6). In its active form, EIF2AK3 can phosphorylate the α-subunit of EIF2A. This phosphorylation is leading to decreased rates of protein synthesis, which further leads to the translation of specific mRNAs, such as ATF4. IRE1α is known to activate the transcription factor XBP1. α-Syn can be released from cells into the extracellular space either as free protein or packed in exosomes formed in multivesicular bodies. ATG5 autophagy-related gene 5, LAMP-2A lysosome-associated membrane protein type 2A, HSC70 the heat shock cognate protein of 70 kDa, EIF2AK3 eukaryotic translation initiation factor 2 alpha kinase 3, EIF2A eukaryotic translation initiation factor 2A, ATF4 activating transcription factor 4, IRE1α inositol requiring enzyme 1α, XBP1 X-box-binding protein 1, ATF6 activating transcription factor 6

This result was unexpected, since we had previously shown that stimulation of autophagy protected from α-Syn-induced toxicity^20^. Hence, we hypothesized that *ATG5* silencing would activate other cellular α-Syn disposal systems to compensate for the lack of autophagy, such as the UPS, chaperones, CMA, UPR, or release of α-Syn into the cell culture medium (Fig. 2).

### No compensatory activation of the UPS upon *ATG5* silencing

The UPS is one major route to degrade preferentially short-lived proteins^16,25^. Therefore, we evaluated the effect of *ATG5* silencing in α-Syn overexpressing cells on the UPS by Western blotting for ubiquitinated proteins in the absence or presence of the proteasome inhibitor MG132.

**Fig. 3:**
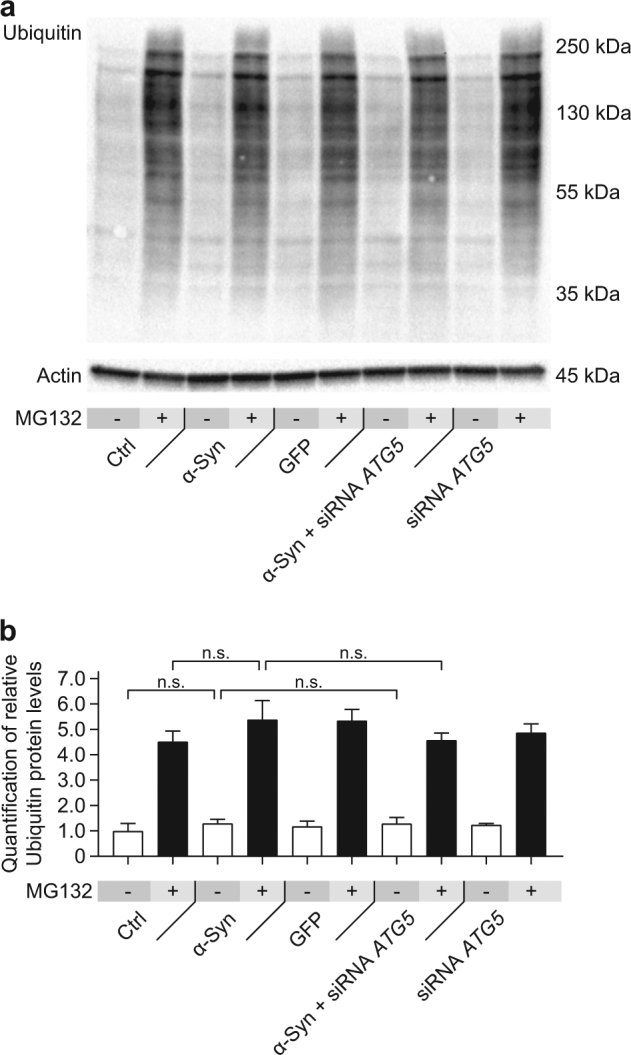
α-Syn overexpression and *ATG5* siRNA do not change ubiquitin levels. The effect of α-Syn overexpression and *ATG5* siRNA on the level of ubiquitinated proteins as global measure for the activity of the ubiquitin–proteasome system was analyzed in the absence and presence of the proteasome inhibitor MG132. **a** Representative Western blot for ubiquitin in naïve control cells (Ctrl), cells overexpressing α-Syn, cells overexpressing the control protein GFP, cells overexpressing α-Syn, and treated with *ATG5* siRNA, and naïve cells treated with *ATG5* siRNA. β-actin was used as loading control. **b** Quantification of ubiquitin, normalized to β-actin, from Western blots as shown in **a**. α-Syn overexpression, GFP overexpression, and *ATG5* knockdown did not alter the global level of ubiquitinated proteins, neither with or without additional proteasome inhibition. Data in **b** are mean ± standard error from *n* = 3 biological replicates. n.s., not significant, one-way analysis of variance with Bonferroni’s post hoc test

Expectedly, 4 h after treatment of the cells with 10 µM MG132, the levels of ubiquitin increased, showing that MG132 effectively inhibited the UPS. However, we did not observe differences in the levels of ubiquitinated proteins between naïve control cells, α-Syn overexpressing cells, GFP overexpressing cells, α-Syn overexpressing cells treated with *ATG5* siRNA, or naïve cells treated with *ATG5* siRNA (Fig. 3a, b).

Furthermore, we did not observe a significant impact of proteasome inhibition on intracellular α-Syn levels alone or in combination with *ATG5* silencing (Supplementary Figure S2).

These results indicate that the UPS was not activated to compensate for the inhibition of autophagy.

### No compensatory increase of chaperone levels upon *ATG5* silencing

Chaperones are ubiquitously expressed to support the physiological folding process of newly synthesized or aberrantly folded proteins^26,27^. Furthermore, chaperones can target irreparably misfolded protein for degradation via the UPS or autophagy^26,27^. Thus, we analyzed the levels of different α-Syn chaperones (heat shock protein 27, HSP27; heat shock protein 70, HSP70; heat shock protein 90, HSP90) in α-Syn overexpressing cells with and without silencing of *ATG5*.

**Fig. 4:**
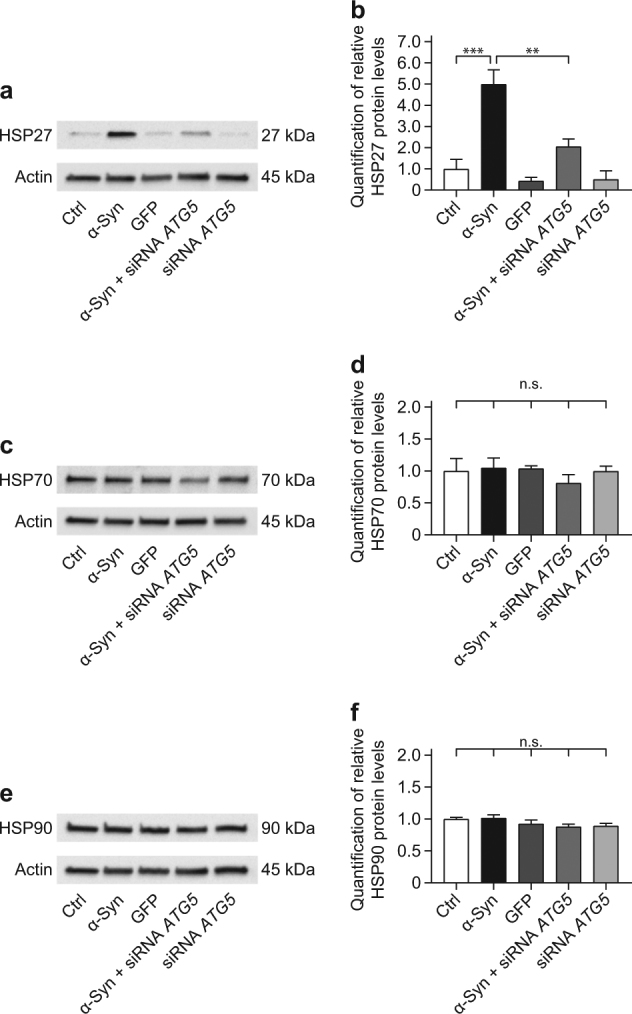
α-Syn-induced increase in HSP27 is prevented by *ATG5* siRNA. The effect of α-Syn overexpression and *ATG5* siRNA on the level of chaperones was analyzed in naïve control cells (Ctrl), cells overexpressing α-Syn, cells overexpressing the control protein GFP, cells overexpressing α-Syn and treated with *ATG5* siRNA, and naïve cells treated with *ATG5* siRNA. β-actin was used as loading control. **a** Representative Western blot for HSP27. **b** Quantification of HSP27, normalized to β-actin, from Western blots as shown in **a**, showing an α-Syn-induced HSP27 increase, which is prevented by *ATG5* siRNA. **c** Representative Western blot for HSP70. **d** Quantification of HSP70, normalized to β-actin, from Western blots as shown in **c**, showing stable expression across the experimental conditions. **e** Representative Western blot for HSP90. **f** Quantification of HSP90, normalized to β-actin, from Western blots as shown in **e**, showing stable expression across the experimental conditions. Data in **b**, **d**, **f** are mean ± standard error from *n* ≥ 3 biological replicates. n.s., not significant, **p < 0.01, ***p < 0.001; one-way analysis of variance with Bonferroni’s post hoc test

HSP27 protein levels were not significantly altered by *ATG5* silencing in naïve cells. However, α-Syn overexpression increased HSP27 levels compared to naïve control, and *ATG5* silencing reduced HSP27 levels in α-Syn overexpressing cells (Fig. 4a, b).

HSP70 (Fig. 4c, d) and HSP90 (Fig. 4e, f) levels were not significantly different between naïve cells, α-Syn overexpressing cells, GFP overexpressing control cells, α-Syn overexpressing cells treated with *ATG5* siRNA, or naïve cells treated with *ATG5* siRNA.

In summary, *ATG5* silencing in α-Syn overexpressing cells did not lead to a compensatory increase of the investigated chaperones.

### No compensatory increase of CMA activity upon *ATG5* silencing

Protein substrates containing a KFERQ-motif can be recognized by a cytosolic chaperone called heat shock cognate protein of 70 kDa (HSC70) and together with co-chaperones dock to the lysosome-associated membrane protein type 2A (LAMP-2A), to be internalized into the lysosome for degradation^28–30^.

**Fig. 5:**
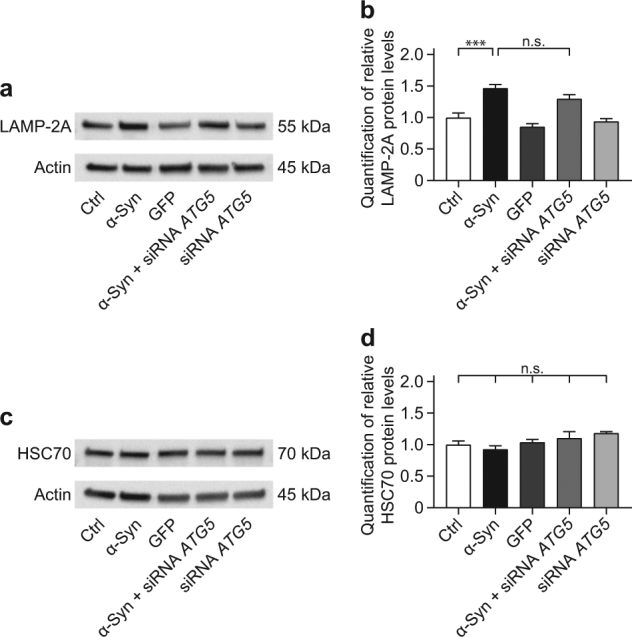
α-Syn-induced increase in LAMP-2A is not changed by *ATG5* siRNA. The effect of α-Syn overexpression and *ATG5* siRNA on markers for chaperone-mediated autophagy was analyzed in naïve control cells (Ctrl), cells overexpressing α-Syn, cells overexpressing the control protein GFP, cells overexpressing α-Syn and treated with *ATG5* siRNA, and naïve cells treated with *ATG5* siRNA. β-actin was used as loading control. **a** Representative Western blot for LAMP-2A. **b** Quantification of LAMP-2A, normalized to β-actin, from Western blots as shown in **a**, showing an α-Syn-induced LAMP-2A increase, which was not changed by *ATG5* siRNA. **c** Representative Western blot for HSC70. **d** Quantification of HSC70, normalized to β-actin, from Western blots as shown in **c**, showing stable expression across the experimental conditions. Data in **b**, **d** are mean ± standard error from n ≥ 3 biological replicates. n.s., not significant, ***p < 0.001; one-way analysis of variance with Bonferroni’s post hoc test

In our cell model, α-Syn overexpression significantly increased LAMP-2A levels compared to naïve control cells, whereas *ATG5* silencing did not alter the increased LAMP-2A levels (Fig. 5a, b).

The HSC70 protein levels did not differ between untransduced and α-Syn overexpressing cells and were not altered by *ATG5* silencing (Fig. 5c, d).

These results suggest that CMA was activated upon α-Syn overexpression. However, there was no indication of a further compensatory increase of CMA activity after *ATG5* silencing.

### No compensatory activation of the UPR upon ATG5 silencing

The UPR is another system to discard misfolded proteins, which is activated upon accumulation of misfolded proteins inside the endoplasmic-reticulum by three different stress sensors: the eukaryotic translation initiation factor 2 alpha kinase 3 (EIF2AK3), inositol requiring enzyme 1α (IRE1α), and activating transcription factor 6 (ATF6). Activated EIF2AK3 phosphorylates the eukaryotic translation initiation factor 2A (EIF2A). IRE1α may activate non-conventional splicing of X-box-binding protein 1 (XBP1)^31–33^. To investigate if the UPR was activated upon inhibition of autophagy by *ATG5* silencing, we quantified important UPR key regulators by Western blotting.

**Fig. 6:**
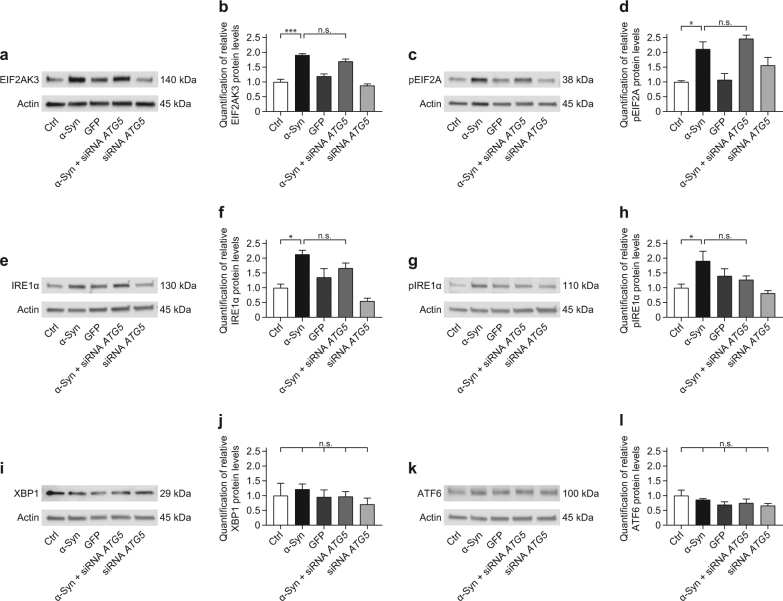
α-Syn-induced increases in EIF2AK3, pEIF2A, IRE1α, and pIRE1α are not changed by *ATG5* siRNA. The effect of α-Syn overexpression and *ATG5* siRNA on markers for unfolded protein response was analyzed in naïve control cells (Ctrl), cells overexpressing α-Syn, cells overexpressing the control protein GFP, cells overexpressing α-Syn and treated with *ATG5* siRNA, and naïve cells treated with *ATG5* siRNA. Representative Western blots for (**a**) the eukaryotic translation initiation factor 2 alpha kinase 3 (EIF2AK3), (**c**) phosphorylated EIF2A (pEIF2A), (**e**) inositol requiring enzyme 1α (IRE1α), (**g**) phosphorylated IRE1α (pIRE1α), (**i**) X-box-binding protein 1 (XBP1), and (**k**) activating transcription factor 6 (ATF6). β-actin was used as loading control. Quantification of (**b**) EIF2AK3, (**d**) pEIF2A, (**f**) IRE1α, (**h**) pIRE1α, (**j**) XBP1, and (**l**) ATF6, showing α-Syn-induced increases in EIF2AK3, pEIF2A, IRE1α, and pIRE1α, which were not significantly changed by *ATG5* siRNA. Data in **b**, **d**, **f**, **h**, **j**, **l**, are mean ± standard error from *n* ≥ 3 biological replicates. n.s., not significant, *p < 0.05, ***p < 0.001; one-way analysis of variance with Bonferroni’s post hoc test

α-Syn overexpression increased EIF2AK3 protein levels, whereas additional *ATG5* silencing did not affect EIF2AK3 levels (Fig. 6a, b). Levels of phosphorylated (i.e. activated) EIF2A (pEIF2A) were elevated in α-Syn overexpressing cells, without further increase by *ATG5* silencing (Fig. 6c, d).

We further investigated the IRE1α/XBP1 branch of the UPR. α-Syn overexpression increased total IRE1α and phosphorylated (i.e. activated) IRE1α (pIRE1α), whereas *ATG5* knockdown had no additional effect (Fig. 6e–h). The protein levels of XBP1 were not altered by α-Syn overexpression or *ATG5* silencing (Fig. 6i, j).

We then investigated the ATF6 branch of the UPR. There was no effect of α-Syn overexpression or *ATG5* silencing on ATF6 levels (Fig. 6k, l).

These data suggest that α-Syn overexpression increased the activity of the EIF2AK3 and IRE1α-dependent branches, leaving the ATF6 branch of the UPR unaltered. Since *ATG5* silencing did not further activate the EIF2AK3 and IRE1α-dependent branches, these mechanisms did not appear to be responsible for the protection against α-Syn-induced toxicity observed after *ATG5* silencing.

### *ATG5* silencing increases α-Syn secretion in the exosomal fraction

Since the intracellular protein degradation machineries did not appear to compensate for the impairment of autophagy in the α-Syn model, we investigated whether *ATG5* silencing influenced the release of α-Syn into the cell culture medium.

**Fig. 7:**
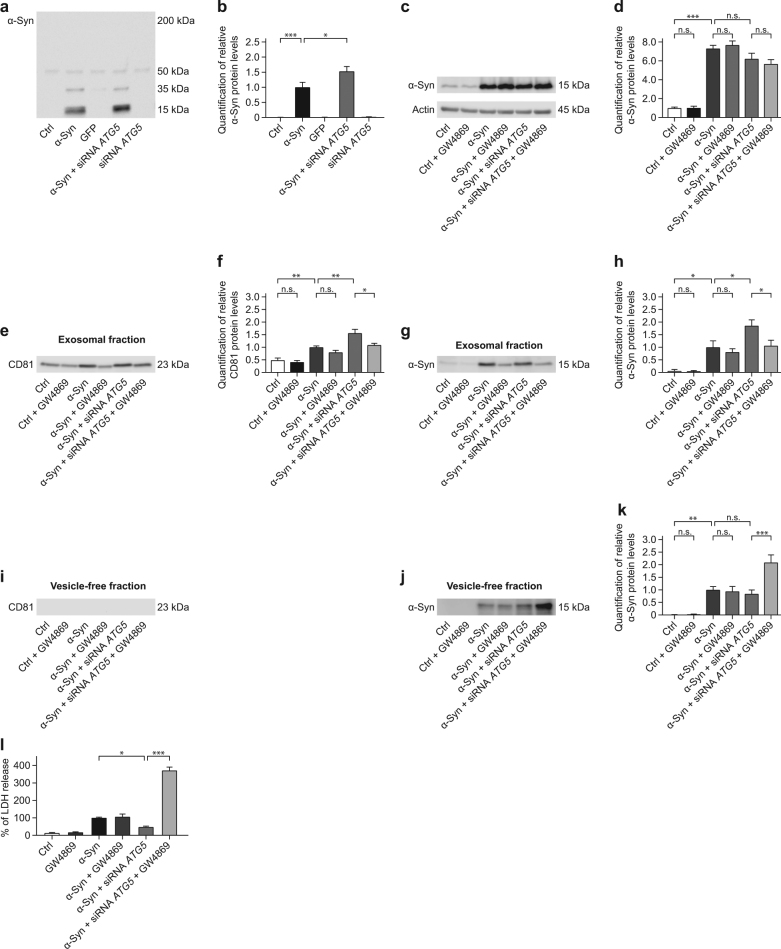
α-Syn-induced exosomal secretion is further increased by *ATG5* siRNA. The effect of α-Syn overexpression and *ATG5* siRNA on the release of α-Syn from cells was analyzed by Western blot in the conditioned medium of naïve control cells (Ctrl), cells overexpressing α-Syn, cells overexpressing the control protein GFP, cells overexpressing α-Syn treated with *ATG5* siRNA, and naïve cells treated with *ATG5* siRNA with or without GW4869 inhibitor. Furthermore, the effect of *ATG5* siRNA combined with exosome inhibition to LDH release was analyzed. **a** Representative Western blot for α-Syn protein in the medium, showing the monomer at 15 kDa and an oligomer at 35 kDa. The faint band at 50 kDa is unchanged across the experimental groups and considered to be nonspecific. **b** Quantification of α-Syn protein (15 kDa band) from Western blots as shown in **a**, showing release of α-Syn from α-Syn overexpressing cells, which is further increased by additional *ATG5* siRNA. **c** Representative Western blot for intracellular α-Syn protein. β-actin was used as loading control. **d** Quantification of α-Syn protein from Western blots as shown in **c**, showing no significant effect of naïve control cells (Ctrl), cells overexpressing α-Syn, and cells overexpressing α-Syn treated with *ATG5* siRNA with GW4869 inhibitor compared to respective control cells. Intracellular levels of α-Syn significantly increased upon overexpression by comparison to naïve cells. **e** Representative Western blot for the exosomal marker CD81 in the vesicle enriched fraction of the medium. **f** Quantification of CD81 protein from Western blots as shown in **c**, showing an increase in exosomes in the medium by *ATG5* siRNA. A further inhibition of exosome biogenesis lead to a decrease in exosomes in the medium by *ATG5* siRNA. **g** Representative Western blot for α-Syn in the vesicle enriched fraction. **h** Quantification of α-Syn protein from Western blots as shown in **g**, showing exosomal secretion of α-Syn from α-Syn overexpressing cells, which is further increased by additional *ATG5* siRNA. Furthermore, an inhibition of exosome biogenesis lead to a decrease in exosomal secretion of α-Syn in the medium by *ATG5* siRNA. **i** Representative Western blot for the exosomal marker CD81, showing the absence of exosomes in the vesicle-free fraction of the medium. **j** Representative Western blot for α-Syn in the vesicle-free fraction. **k** Quantification of α-Syn protein from Western blots as shown in **j**, showing release of free α-Syn from α-Syn overexpressing cells, which is not altered by additional *ATG5* siRNA. Inhibition of exosome biogenesis together with inhibition of autophagosome formation by *ATG5* siRNA increased release of free α-Syn in the medium. **l** Quantification of lactate dehydrogenase (LDH) released into the culture medium. Data are expressed as percentage of α-Syn. Naïve control cells and α-Syn overexpressing cells treated with GW4869 did not show elevated toxicity levels compared to respective control cells. *ATG5* silencing combined with GW4869 treatment significantly increased α-Syn-induced toxicity. Data in **b**, **d**, **f**, **h**, **k**, **l** are mean ± standard error from *n* ≥ 3 biological replicates. n.s., not significant, *p < 0.05, **p < 0.01, ***p < 0.001; one-way analysis of variance with Bonferroni’s post hoc test

In the medium of naïve cells, GFP overexpressing cells and *ATG5* knockdown cells, α-Syn was not detectable by Western blot (Fig. 7a, b). Four days of α-Syn overexpression, however, led to detectable α-Syn levels in the medium (Fig. 7a, b). Interestingly, silencing of *ATG5* in α-Syn overexpressing cells further increased the release of α-Syn (Fig. 7a, b). Thus, LUHMES neurons appear to increase α-Syn release as a compensatory mechanism to reduce the intracellular α-Syn burden if degradation by autophagy is blocked.

We then investigated whether α-Syn is released as free protein (either actively or passively through leakage as a result of increased toxicity) or via vesicular release packed in exosomes. Therefore, we fractionated the medium by ultracentrifugation into vesicle-free and exosomal fractions.

To block exosome generation, we used GW4869, a neutral sphingomyelinase (nSMase) inhibitor that blocks the ceramide-mediated inward budding of multivesicular bodies (MVBs) and thus the release of mature exosomes from MVBs, in all subsequent experiments^34,35^.

First, we investigated the intracellular load of α-Syn upon overexpression of α-Syn and overexpression of α-Syn combined with *ATG5* silencing, with and without GW4869 treatment. Expectedly, intracellular levels of α-Syn significantly increased upon overexpression by comparison to naïve cells. However, neither *ATG5* silencing nor GW4869 treatment significantly affected these levels (Fig. 7c, d).

Secondly, the exosomal fractions were investigated. The exosomal marker CD81 was used as a quality control of the fractionation procedure and was present in all the exosomal samples. Furthermore, GW4869 systematically led to decreased CD81 levels, indicating that exosomal secretion was indeed affected by this treatment. α-Syn overexpression alone significantly increased CD81 levels in the medium. Moreover, α-Syn overexpression combined with *ATG5* silencing led to a further increase in CD81 levels, demonstrating elevated exosomal secretion (Fig. 7e, f). Expectedly, inhibition of exosome generation with GW4869 reduced the increased exosomal secretion followed by *ATG5* silencing in α-Syn overexpression (Fig. 7e, f). Moreover, by inhibiting exosome biogenesis with GW4869 we detected a slight, however not significant decrease of ALIX and Flotilin-1 levels in naïve cells, α-Syn overexpressing cells and α-Syn overexpressing cells treated with siRNA against *ATG5* compared to cells of the same conditions not treated with GW4869 (Supplementary Figure S3a, b, d, e).

Furthermore, α-Syn levels in the exosomal fraction were increased in α-Syn overexpressing cells compared to naïve cells, and even further increased by additional *ATG5* silencing (Fig. 7g, h), thus corresponding to CD81 levels. This suggests that blockage of autophagy by *ATG5* silencing increased the secretion of α-Syn in the exosomal fraction as an alternative mechanism to autophagy to reduce the cellular α-Syn burden (Fig. 7g, h). Accordingly, treatment with GW4869 also decreased α-Syn levels in the exosomal fraction followed by *ATG5* silencing (Fig. 7g, h).

Subsequently, the vesicle-free medium fraction was analyzed. The exosomal markers CD81, ALIX, and Flotilin-1 were absent in all the exosome-depleted samples (Fig. 7i and Supplementary Figure S3c, f). α-Syn levels in the vesicle-free fraction of the medium were increased by overexpression of α-Syn as compared to the vesicle-free fraction of the medium from naïve cells, but *ATG5* silencing had no further impact on these levels. Strikingly, a combination of α-Syn overexpression, *ATG5* silencing, and GW4869 led to a marked increase of α-Syn in the vesicle-free fraction compared to α-Syn overexpression alone, α-Syn overexpression and *ATG5* silencing without GW4869 treatment, or α-Syn overexpression and GW4869 treatment without *ATG5* silencing (Fig. 7j, k). This suggests that α-Syn was released in a vesicle-free manner if autophagy and exosomal secretion were impaired.

We excluded the enrichment of other types of vesicles, such as lysosomes (LAMP1) and autophagosomes (p62) in both the exosomal and the vesicle-free fractions (Supplementary Figure S3g, h, i, j).

We then investigated cytotoxicity by measuring LDH released into the cell culture medium (Fig. 7l). GW4869 had no effect on naïve control cells, α-Syn overexpressing cells, α-Syn overexpressing cells transfected with *GAPDH* siRNA, or cells overexpressing GFP (Fig. 7l and Supplementary Figure 3k). However, blockage of exosomal secretion with GW4869 treatment dramatically increased toxicity in cells overexpressing α-Syn in which also autophagy was blocked by *ATG5* silencing (Fig. 7l and Supplementary Figure S3k).

This suggests that increased α-Syn released in the exosomal fraction upon blockade of autophagy is a vital rescue mechanism. When this rescue mechanism was blocked by GW4869, increased toxicity was observed, which is highly consistent with the striking increase in α-Syn levels in the vesicle-free medium under the same conditions (Fig. 7j, k).

## Discussion

In LUHMES neurons α-Syn is degraded by autophagy and activation of autophagy protects them against α-Syn-induced toxicity^20^. Dysfunction of the autophagy-lysosomal pathway is considered to play an essential role in hereditary and sporadic forms of PD. In the present study we inhibited autophagy at the earliest stage, i.e. the formation of the phagophore^21^ by silencing of *ATG5*, a key player in phagophore formation. Unexpectedly, we found that also inhibition of autophagy by silencing of *ATG5* protected LUHMES neurons from α-Syn-induced toxicity. Thus, we hypothesized that the neurons would activate a compensatory mechanism to dispose of α-Syn. Therefore, we studied the mechanisms neurons are most likely to initiate to compensate for impaired autophagy, when there is a need to cope with an excess of intracellular α-Syn. The effect of *ATG5* silencing on UPS, chaperones, CMA, UPR, and the release of α-Syn into the extracellular space were investigated (Fig. 2). While there was no evidence for a compensatory upregulation of the intracellular elimination pathways, *ATG5* silencing increased exosomal secretion of α-Syn. Furthermore, the combination of inhibition of both autophagy and exosomal secretion substantially exacerbated α-Syn-induced toxicity. These findings demonstrate that exosomal secretion of α-Syn is a functionally relevant rescue mechanism for impaired intracellular autophagy.

The role of the UPS in α-Syn degradation is still controversial. Some reports show no effect of α-Syn overexpression on ubiquitin levels^36^ and no accumulation of α-Syn after UPS inhibition^37,38^, whereas others show that α-Syn is degraded by the UPS^14,39^. Notwithstanding, we did not observe any alterations in ubiquitin levels with or without proteasome inhibition, when α-Syn was overexpressed and *ATG5* was silenced. Moreover, proteasome inhibition alone or with additional *ATG5* silencing had also no effect on intracellular α-Syn levels. This suggests that the UPS was not functionally compensating α-Syn degradation after blocking autophagy.

Molecular chaperones are also thought to protect cells against misfolded proteins^27,40,41^. Specifically, HSP27 was found to be elevated on mRNA and protein levels in postmortem brains affected by α-Syn pathology. Moreover, HSP27 overexpression protected against α-Syn-induced toxicity in vitro^42,43^. Consistently, we found elevated HSP27 protein levels upon α-Syn overexpression. However, additional *ATG5* silencing led to a reduction rather than a further increase of HSP27 levels. This suggests that HSP27 was not responsible for the attenuation of α-Syn overexpression-mediated toxicity. The levels of HSP70 and HSP90, which play a role in the pathogenesis of synucleinopathies and are present in Lewy bodies^44^, were not altered in our model. Thus, these α-Syn-linked chaperones were not upregulated as compensatory mechanism upon inhibition of autophagy in our model. Since we did not modulate HSP27, HSP70, or HSP90 expression, we cannot conclude on the contribution of these molecular chaperones to α-Syn degradation if autophagy is intact. However, investigating this would have gone beyond the scope of this work.

CMA is also implicated in the degradation of α-Syn^15^. In α-Syn transgenic mice mRNA levels of the CMA marker LAMP-2A were increased^45^. Consistently, we found elevated LAMP-2A protein levels upon α-Syn overexpression. However, *ATG5* silencing did not further elevate LAMP-2A levels. Also, the levels of the CMA marker HSC70 were unaltered between the experimental conditions. Even though we cannot conclude on the contribution of the CMA to α-Syn degradation in our model under basal conditions, our data suggest that the activity of the CMA was not increased, if autophagy was blocked and thus did not appear to compensate for inhibition of autophagy.

The accumulation of aberrant proteins may also activate the UPR via three stress sensors: EIF2AK3, IRE1α, and ATF6^32,33^. Levels of pEIF2A, a substrate of EIF2AK3, were elevated in the substantia nigra of PD patients and rats overexpressing α-Syn^46^. Consistently, we found elevated EIF2AK3 and pEIF2A levels. Also, IRE1α and pIRE1α levels were elevated in our model, suggesting that the UPR was activated upon α-Syn overexpression. However, there was no further compensatory elevation of these markers after *ATG5* silencing. ATF6 and XPB1 levels were not altered. This is consistent with observations in α-Syn overexpressing rats^47^ and rat brain slices^48^. Although we did not directly measure the activity of the UPR, the fact that UPR markers were not altered upon autophagy blockage, suggests that the UPR was not activated as compensatory mechanism after autophagy inhibition.

Our results demonstrate a significant increase in CD81-positive extracellular vesicles (EVs) in α-Syn-transduced neurons as compared to naïve control cells. Transfection with *ATG5* siRNA further increased the amount of CD81 in the extracellular vesicle-containing fraction (Fig. 7e, f). These results thus suggest that silencing *ATG5* enhances exosomal secretion in α-Syn overexpressing neurons. Comparing the amount of ALIX and Flotilin-1 showed a similar trend, although this effect did not reach statistical significance. Given the heterogeneity of EVs^49^, tetraspanins and other EV-associated proteins (e.g. ALIX or Flotilin-1) likely label different EV subpopulations which vary in size, intracellular origin, and protein composition, and thus might account for the differences observed with CD81 and ALIX or Flotilin-1.

The mechanism that controls the release of distinct exosomal subpopulations are not well understood thus making it difficult to block the release of all exosome subtypes simultaneously. Here, we used the nSMase inhibitor GW4869, that inhibits the ceramide-mediated inward budding of MVBs^50,51^. However, not all exosome subtypes are generated in a ceramide-dependent manner^52^. Instead, some exosome subpopulations may be generated through a mechanism involving the endosomal sorting complexes required for transport-(ESCRT) pathway independent from ceramide^53^. The ESCRT is a protein complex that plays a vital role in a number of cellular processes including MVB biogenesis. Therefore, it is not surprising that GW4869 does not induce an overt change in the overall amount of released exosomes (Fig. 7e, f), although treatment with GW4869 did decrease the amount of CD81-positive vesicles in the exosomal fraction in α-Syn overexpressing cells combined with *ATG5* silencing. In accordance with the different exosome generation mechanisms outlined above, ceramide-dependent exosome generation and secretion may be more relevant under conditions where α-Syn is overexpressed and autophagy is blocked by *ATG5* silencing, thus accounting for a significant effect of GW4869 under these conditions (Fig. 7e, f).

Interestingly, *ATG5* silencing increased α-Syn release in the vesicle-enriched (exosomal) fraction of the medium, whereas α-Syn levels in the vesicle-free fraction were not altered. The inhibition of exosome generation alone had no effect on cell viability in the α-Syn overexpressing group. This suggests that this form of α-Syn disposal does not contribute significantly in alleviating the α-Syn burden, as long as the autophagy machinery remains intact. However, upon blockage of autophagy, exosomal secretion of α-Syn appears to become vital for the cells.

Previous studies had investigated the consequences of impairment of autophagosome–lysosome fusion by bafilomycin A1 (BafA1). In line with our findings, BafA1 also increased vesicular α-Syn release^54,55^. However, BafA1 increased α-Syn-induced toxicity^55,56^. Consistently, we have also reported that blocking autophagosome–lysosome fusion by chloroquine exacerbates α-Syn-induced toxicity in our model^20^. Since the EVs after BafA1 treatment appeared to be derived from MVBs that had interacted or were in contact with autophagolysosomes^55^, this mechanism of vesicular release is most likely different from the form of exosomal secretion we observed after inhibition of autophagosome formation.

Thus, it appears that impaired lysosomal degradation of α-Syn within autophagosomes is detrimental for neurons. However, bypassing the autophagy machinery completely, by very upstream inhibition of autophagosome formation (e.g. *ATG5*-silencing) seems to lead to a protective form of exosomal α-Syn secretion.

Previous reports showed that vesicular α-Syn contributes to cell-to-cell spreading of α-Syn pathology and induced toxicity in recipient cells^54^. In contrast, others have reported that vesicle-free extracellular α-Syn rather than exosomal α-Syn would contribute to toxicity in recipient cells^57^. Thus, the question whether vesicular or vesicle-free α-Syn is more detrimental for cells still remains elusive. One possible interpretation is that different ways of exosomal α-Syn secretion exist. On the one hand, autophagosome-derived vesicles seem to be deleterious by facilitating cell-to-cell spreading^55^. On the other hand, the exosomal α-Syn secretion we observed upon upstream bypassing of the autophagosome protected the cells against α-Syn-induced toxicity.

In summary, we found that inhibition of phagophore formation by *ATG5* silencing, i.e. a very early step in autophagy, protected LUHMES neurons against cell death induced by the overexpression of human wild-type α-Syn. Moreover, we found that the cells utilized exosomal secretion of α-Syn as compensatory mechanism to discard α-Syn. Additionally, we demonstrated a synergistic detrimental effect of impaired autophagy and impaired exosomal secretion. These cellular mechanisms appear relevant for the development of disease-modifying therapies for PD patients suffering from dysfunctions in the autophagy pathway.

## Materials and methods

### Cell culture

LUHMES cells^58^ were cultured on 0.1 mg/mL poly-l-lysine (Sigma-Aldrich, St. Louis, MO, USA) coated flasks (Nunclon DELTA surface, NUNC A/S, Roskilde, Denmark) in growth medium consisting of DMEM/F12 (Sigma-Aldrich) with 1% N_2_ supplement (Life Technologies, Carlsbad, CA, USA), and 0.04 µg/mL basic fibroblast growth factor (PeproTech, Rocky Hill, CT, USA). For experiments, cells were plated on multi-well plates, in differentiation medium consistent of DMEM/F12 with 1% N_2_ supplement, 1 µg/mL tetracycline, 0.49 µg/mL dibutyryl cyclic AMP (Sigma-Aldrich), and 2 ng/mL glial cell-derived neurotrophic factor (GDNF; R&D Systems, Minneapolis, MN, USA). Before plating, the multi-well plates were coated with 0.1 mg/mL poly-l-lysine (Sigma-Aldrich) at 4 °C overnight, washed three times with phosphate-buffered saline (PBS; Life Technologies) followed by coating with 5 µg/mL bovine fibronectin (Sigma-Aldrich) at 37 °C and 5% CO_2_ overnight, followed by once washing with PBS (Life Technologies), and air-drying. LUHMES cells were cultured at 37 °C, 5% CO_2_, and water-saturated air.

### Transduction with adenoviral vectors

48 h after plating, transduction with adenoviral vectors was performed with a multiplicity of infection (MOI) of 2. As previously described^20,59^ we used adenoviral vectors, expressing human wild-type α-Syn, or green fluorescent protein (GFP) under a cytomegalovirus promotor (BioFocus DPI, Leiden, Netherlands). In untransduced cells, fresh medium without adenoviral vectors was added. To remove remaining adenoviral particles, the cells were rinsed three times with PBS 24 h after transduction and fresh differentiation medium was added.

### Small interfering RNA transfection

Transfection with small interfering RNAs (siRNA) was performed 1 day following the adenoviral transduction after removal of remaining viral vectors. siRNAs (Stealth RNAi, Thermo Fisher Scientific) against *ATG5* or *GAPDH*, as well as Lipofectamine RNAiMAX (Thermo Fisher Scientific) were diluted in Opti-Mem (Thermo Fisher Scientific) medium separately. For complexation of the siRNA with Lipofectamine RNAiMAX, both dilutions were mixed, gently shaken, and incubated for 20 min. Thereafter, the siRNA-Opti-Mem-Lipofectamine RNAiMAX mix was added to the cells with final concentrations of 50 nM of the siRNA and 2 µl/mL of Lipofectamine RNAiMAX, respectively.

### Treatment of LUHMES cells with different compounds

To investigate LC3B-II, a marker for autophagosomes, untransduced cells were transfected with the siRNA against *ATG5* 72 h after plating as described above. Forty-eight hours after the transfection the cells were treated with 50 µM chloroquine (Sigma-Aldrich), or the respective amount of medium in controls to determine if alterations of LC3B-II levels were caused by increased autophagic flux or impaired degradation of autophagosomes. After 24 h the cells were harvested for Western blot analysis.

For the quantification of ubiquitin levels to monitor the activity of the UPS, LUHMES cells were cultured and transduced as described above. On day 4 after transduction, the cells were treated with 10 µM of the proteasome inhibitor MG132 (Tocris Bioscience, Bristol, United Kingdom) 4 h prior to harvesting the cells for Western blot analysis.

To inhibit exosomal release 1 day after transduction, the cells were treated with 5 µM of the nSMase inhibitor GW4869 (Sigma-Aldrich).

### Measurement of cytotoxicity

The fluorescence-based CytotoxONE Membrane Integrity Assay (Promega, Fitchburg, WI, USA) was performed to measure lactate dehydrogenase (LDH) released into culture medium from dying cells. The assay was performed 6 days after virus transduction according to the manufacturer’s instructions. Fluorescence levels were determined with a fluorescence microplate reader (FLUOstar Omega, BMG LABTECH, Ortenberg, Germany).

### Real-time quantitative reverse transcription polymerase chain reaction (qRT-PCR)

Cells were harvested 6 days after adenoviral transduction using the RLT Plus-lysis buffer from the RNeasy Mini Kit (Qiagen, Venlo, Netherlands), supplemented with β-mercaptoethanol (Sigma-Aldrich) to deactivate RNAases. RNA extraction was performed according to the instructions of the RNeasy Mini Kit manual. The total mRNA concentration was determined spectrophotometrically using the Nanodrop 2000c (Thermo Fisher Scientific). One µg of the mRNA of each sample was reversely transcribed into total cDNA using the iScript reverse transcription supermix (Bio-Rad Laboratories, Hercules, California, CA, USA). For real-time qRT-PCR, the StepOnePlus real-time PCR system (Life Technologies) was used. The comparative threshold cycle (Ct) was analyzed and qRT-PCRs were conducted in three independent experiments using the TaqMan Universal Master Mix II with UNG (Life Technologies) and the respective TaqMan gene expression assay kit (Life Technologies). The following gene expression assay kits (primer/probe sets) were used: ATG5, assay ID Hs00169468_m1; PPIA, assay ID Hs04194521_s1; PPIB, assay ID Hs00168719_m1; PSMC1, assay ID Hs02386942_g1 (Life Technologies). For quantification, the relative quantities of the target mRNA were normalized to mRNA level in naïve control cells.

### Western blot

Cells were lysed using M-PER protein extraction buffer (Thermo Scientific Pierce Protein Research Products, Rockford, IL, USA) complemented with protease inhibitor cocktail and phosphatase inhibitor cocktail (both from F. Hoffmann-La Roche, Basel, Switzerland) as previously described^20^. After centrifugation at 13,000 × *g* for 10 min at 4 °C, the protein concentration of the supernatant was determined using the BCA protein assay kit (Thermo Scientific Pierce Protein Research Products). Twenty micrograms of each sample were loaded with Roti-Load 1 (Carl Roth, Karlsruhe, Germany) to 4–20% TGX Precast protein gels (Bio-Rad Laboratories). The proteins were then separated by sodium dodecyl sulfate polyacrylamide gel electrophoresis and transferred onto a polyvinylidene fluoride membrane (Bio-Rad Laboratories). Membranes were blocked in Roti-Block (10×; Carl Roth) diluted 1:10 in milliQ water for at least 1 h at room temperature. Thereafter the membranes were incubated overnight at 4 °C with the respective primary antibody diluted in Tris buffered saline containing 0.05% Tween-20 (TBS-T, pH 7.4) and 10% of Roti-Block (10×). The following primary antibodies were used: rabbit anti-APG5L/ATG5 (1:1000, Abcam, Cambridge, United Kingdom), rabbit anti-LC3B (1:1000; Cell Signaling Technology, Danvers, MA, USA), rabbit anti-LAMP-2A (1:1000, Abcam), rabbit anti-HSC70 (1:500, GeneTex, Irvine, California, CA, USA), mouse anti-HSP27 (1:1000; Cell Signaling Technology), rabbit anti-HSP70 (1:1000; Cell Signaling Technology), rabbit anti-HSP90 (1:1000; Cell Signaling Technology), mouse anti-Ubiquitin (1:500, Santa Cruz Biotechnology, Dallas, TX, USA), rabbit anti-EIF2AK3 (1:1000; Cell Signaling Technology), rabbit anti-pEIF2A (1:1000; Cell Signaling Technology), rabbit anti-IRE1α (1:1000; Cell Signaling Technology), rabbit anti-pIRE1α (1:1000; Novus Biologicals Littleton, Colorado, CO, USA), rabbit anti-ATF6 (1:500; GeneTex, Irvine, California, CA, USA), rabbit anti-XBP-1 (1:500; Santa Cruz Biotechnology), rabbit anti-α-Syn (1:1000; Cell Signaling Technology), mouse anti-CD81 (1:1000; Santa Cruz Biotechnology), rabbit anti-AIP1/ALIX (1:1000; Merck Millipore, Billerica, Massachusetts, MA, USA), mouse anti-Flotilin-1 (1:1000, BD Biosciences, San Jose, CA, USA), rabbit anti-LAMP1 (1:1000, Cell Signaling Technology), mouse anti-p62 (1:1000, Merck Millipore), and rabbit anti-β-actin (1:2000; Cell Signaling Technology). After washing three times with TBS-T the membranes were incubated for 2 h at room temperature with the respective horseradish peroxidase-conjugated secondary antibodies (anti-rabbit IgG, 1:5000 [PI-1000]; anti-mouse IgG, 1:2500 [PI-2000]; Vector Laboratories, Burlingame, CA, USA). The bound antibodies were visualized using Clarity Western ECL Substrate (Bio-Rad Laboratories). Images were taken with the Odyssey Fc (LI-COR Biotechnology, Lincoln, Nebraska, NE, USA) imaging system. Optical densities of Western blot bands were quantified by measuring integrated density using the Fiji software. All data were normalized to the density of the respective β-actin band. For each condition, at least three independent experiments were performed.

### Investigation of the cell culture medium

To detect α-Syn released into the cell culture medium, the medium was centrifuged at 988 × *g* for 15 min to remove cell debris. The medium was then concentrated for Western Blot analysis with a 3 kDa molecular weight cut-off filter (Vivaspin 6; Sartorius, Göttingen, Germany). The protein content in the medium was quantified using the Commassie Plus Protein Assay Reagent (Thermo Fisher Scientific). Fifty micrograms of protein were loaded per lane on a 4–20% TGX Precast protein gel (Bio-Rad Laboratories). Western Blot analysis was performed as described except for fixation of the membranes with PBS containing 0.37% formaldehyde (Sigma-Aldrich) for 30 min at room temperature. After three times washing with PBS, the membranes were blocked as described above. For the analysis of the exosome and vesicle-free fraction, the supernatant from the cells was centrifuged at 1000 × *g* for 10 min at 4 °C. Then, the supernatant was transferred to an ultracentrifugation tube (Beckman Coulter, Brea, CA, USA) and centrifuged at 100,000 × *g* for 90 min at 4 °C in an Optima MAX-XP ultracentrifuge (Beckman Coulter). The fraction containing the pellet with the exosomes was washed once with PBS and again centrifuged at 100,000×*g* for 90 min at 4 °C. Then the pellet was resuspended in radioimmunoprecipitation assay (RIPA) buffer (Sigma-Aldrich) followed by shaking for 30 min at 4 °C. The protein content was determined using the BCA protein assay kit as described above. The vesicle-free fraction was concentrated with a molecular weight cut off 3 kDa filter and the protein content was quantified using the Commassie Plus Protein Assay Reagent as described above. For gel electrophoresis equal amounts of protein of each sample were loaded. Due to absence of cells in the medium fraction, no actin loading control was performed. Western blot bands were quantified as described above. For quantification, the relative densities of the Western blot bands were normalized to the mean densities measured in α-Syn overexpressing cells.

### Statistical analysis

Statistical analysis was performed using GraphPad Prism 7.01 (GraphPad Software, La Jolla, CA, USA). All data shown in the figures are presented as mean ± standard error of the mean (SEM). All data were analyzed via one-way ANOVA followed by Bonferroni’s post hoc test, *p*-values < 0.05 were considered as statistically significant.

## Electronic supplementary material

Supplementary Figure S1

Supplementary Figure S2

Supplementary Figure S3

Figure legends for Supplementary Figure S1-S3
