# Supplementary material for: Exosomal secretion of α-synuclein as protective mechanism after upstream blockage of macroautophagy
Source: Cell Death Dis. 2018 Jul 9;9(7):757. doi: 10.1038/s41419-018-0816-2 (PMC6037700; doi:10.1038/s41419-018-0816-2)
Supplement: Supplementary file 4 — Figure legends for Supplementary Figure S1-S3 [file 41419_2018_816_MOESM4_ESM.docx]

**Supplementary Figure S1. Effect of *ATG5* silencing on intracellular α-Syn levels in the presence or absence of chloroquine.**

(a) Representative Western blot with an antibody against α-Syn of naïve control cells (Ctrl), α-Syn overexpressing cells, α-Syn overexpressing cells transfected with a siRNA against *ATG5,* naïve cells transfected with a siRNA against *ATG5*, and cells overexpressing GFP as control protein with (+) or without (-) chloroquine (Chl) treatment. β-actin was used as loading control.

(b) Quantification of the α-Syn band, normalized to β-actin, from Western blots as shown in (a). Chloroquine treatment did not alter intracellular α-Syn levels of naïve control cells, α-Syn overexpressing cells, α-Syn overexpressing cells transfected with a siRNA against *ATG5*, naïve cells transfected with a siRNA against *ATG5*, and GFP overexpressing cells.

Data in (b) are presented as mean ± standard error from n = 3 biological replicates. n.s., not significant, ***p* < 0.01, ****p* < 0.001; one-way analysis of variance with Bonferroni’s *post hoc* test.

**Supplementary Figure S2. Effect of *ATG5* silencing on intracellular α-Syn levels in the presence or absence of proteasome inhibition with MG132.**

(a) Representative Western blot with an antibody against α-Syn of naïve control cells (Ctrl), α-Syn overexpressing cells, cells overexpressing GFP as control protein, α-Syn overexpressing cells transfected with a siRNA against *ATG5,* and naïve cells transfected with a siRNA against *ATG5* with (+) or without (-) MG132 treatment. β-actin was used as loading control.

(b) Quantification of the α-Syn band, normalized to β-actin, from Western blots as shown in (a). Proteasome inhibition had no influence on α-Syn levels of naïve cells, α-Syn overexpressing cells, GFP overexpressing cells, α-Syn overexpressing cells with *ATG5* silencing, or naïve cells with *ATG5* silencing.

Data in (b) are presented as mean ± standard error from n = 3 biological replicates. n.s., not significant, ****p* < 0.001; one-way analysis of variance with Bonferroni’s *post hoc* test.

**Supplementary Figure S3. Investigation of vesicular markers in conditioned medium and the effect of exosome inhibition on LDH release**

The impact of *ATG5* silencing on exosomes, lysosomes and autophagosomes was monitored in the presence and absence of GW4869. This was analyzed by Western blot of naïve control cells (Ctrl), α-Syn overexpressing cells, and α-Syn overexpressing cells transfected with a siRNA against *ATG5* to block autophagy.

(a) Representative Western blot with an antibody against the exosomal marker ALIX of naïve control cells (Ctrl), α-Syn overexpressing cells, and α-Syn overexpressing cells transfected with a siRNA against *ATG5,* with or without GW4869 treatment in the vesicle enriched fraction of the medium.

(b) Quantification of the ALIX protein band from Western blots as shown in (a), indicate a slight increase in the amount of exosomes in the medium from α-Syn overexpressing cells upon *ATG5* silencing.

(c) Representative Western blot with an antibody against the exosomal marker ALIX of naïve control cells (Ctrl), α-Syn overexpressing cells, and α-Syn overexpressing cells transfected with a siRNA against *ATG5,* with or without GW4869 treatment from the vesicle free fraction of the medium, showing the absence of ALIX^+^ vesicles in the vesicle-free fraction of the medium.

(d) Representative Western blot with an antibody against the exosomal marker Flotilin-1 of naïve control cells (Ctrl), α-Syn overexpressing cells, and α-Syn overexpressing cells transfected with a siRNA against *ATG5,* with or without GW4869 treatment in the vesicle enriched fraction of the medium.

(e) Quantification of the Flotilin-1 protein band from Western blots as shown in (d), indicate a slight increase in the amount of exosomes in the medium from α-Syn overexpressing cells upon *ATG5* silencing.

(f) Representative Western blot with an antibody against the exosomal marker Flotilin-1 of naïve control cells (Ctrl), α-Syn overexpressing cells, and α-Syn overexpressing cells transfected with a siRNA against *ATG5,* with or without GW4869 treatment in the vesicle-free fraction of the medium showing the absence of Flotilin-1^+^ vesicles in the vesicle-free fraction of the medium.

(g) Representative Western blot with an antibody against the lysosomal marker LAMP1 of naïve control cells (Ctrl), α-Syn overexpressing cells, and α-Syn overexpressing cells transfected with a siRNA against *ATG5,* with or without GW4869 treatment showing the absence of lysosomes in the vesicle enriched fraction of the medium.

(h) Representative Western blot with an antibody against the lysosomal marker LAMP1 of naïve control cells (Ctrl), α-Syn overexpressing cells, and α-Syn overexpressing cells transfected with a siRNA against *ATG5,* with or without GW4869 treatment showing the absence of lysosomes in the vesicle-free fraction of the medium.

(i) Representative Western blot with an antibody against the autophagosomal marker p62 of naïve control cells (Ctrl), α-Syn overexpressing cells, and α-Syn overexpressing cells transfected with a siRNA against *ATG5,* with or without GW4869 treatment showing the absence of autophagosomes in the vesicle enriched fraction of the medium.

(j) Representative Western blot with an antibody against the autophagosomal marker p62 of naïve control cells (Ctrl), α-Syn overexpressing cells, and α-Syn overexpressing cells transfected with a siRNA against *ATG5,* with or without GW4869 treatment showing the absence of autophagosomes in the vesicle-free fraction of the medium.

(k) Quantification of lactate dehydrogenase (LDH) released into the culture medium as measure for toxicity. Data are expressed as percentage LDH released from α-Syn overexpressing cells. Blockage of exosomal release with GW4869 did not increase toxicity in naïve control cells (Ctrl), α-Syn overexpressing cells, α-Syn overexpressing cells transfected with a siRNA against *GAPDH*, or cells overexpressing GFP as control protein treated with GW4869. In α-Syn overexpressing cells with blockage of autophagy by *ATG5* silencing, however, GW4869 led to significant toxicity, suggesting that exosomal release of α-Syn was vital, upon blockage of autophagy

Data in (b, e, k) are mean ± standard error from n ≥ 3 biological replicates. n.s., not significant, **p* < 0.05, ****p* < 0.001; one-way analysis of variance with Bonferroni’s *post hoc* test.
